# Andrographolide: A Herbal-Chemosynthetic Approach for Enhancing Immunity, Combating Viral Infections, and Its Implication on Human Health

**DOI:** 10.3390/molecules26227036

**Published:** 2021-11-21

**Authors:** Archana Mishra, Haq Abdul Shaik, Rakesh Kumar Sinha, Bakht Ramin Shah

**Affiliations:** 1 South Bohemian Research Center of Aquaculture and Biodiversity of Hydrocenoses, Faculty of Fisheries and Protection of Waters, Institute of Aquaculture and Protection of Waters, University of South Bohemia in České Budějovice, Na Sádkách 1780, 37005 České Budějovice, Czech Republic; bshah@frov.jcu.cz; 2 Institute of Entomology, Biology Centre, Czech Academy of Science, 37005 České Budějovice, Czech Republic; haq@entu.cas.cz; 3 Department of Parasitology, Faculty of Science, University of South Bohemia, 37005 České Budějovice, Czech Republic; 4 Institute of Plant Genetics, Polish Academy of Sciences, 34 Strzeszynska Street, 60-479 Poznan, Poland; sinharakesh_2011@hotmail.com

**Keywords:** *Andrographis paniculata* (AP), andrographolide (AGL), anti-manic, anti-microbial, COVID-19 epidemic, immune booster, herbal-chemo remedy

## Abstract

Plants consistently synthesize and accumulate medically valuable secondary metabolites which can be isolated and clinically tested under in vitro conditions. An advancement with such important phytochemical production has been recognized and utilized as herbal drugs. Bioactive andrographolide (AGL; C_20_H_30_O_5_) isolated from *Andrographis paniculate* (AP) (Kalmegh) is a diterpenoid lactones having multifunctional medicinal properties including anti-manic, anti-inflammatory, liver, and lung protective. AGL is known for its immunostimulant activity against a variety of microbial infections thereby, regulating classical and alternative macrophage activation, Ag-specific antibody production during immune disorder therapy. In vitro studies with AGL found it to be effective against multiple tumors, neuronal disorders, diabetes, pneumonia, fibrosis, and other diverse therapeutic misadventures. Generally, virus-based diseases like ZIKA, influenza A virus subtype (H1NI), Ebola (EBOV), Dengue (DENV), and coronavirus (COVID-19) epidemics have greatly increased scientific interest and demands to develop more effective and economical immunomodulating drugs with minimal side effects. Trials and in vitro pharmacological studies with AGL and medicinally beneficial herbs might contribute to benefit the human population without using chemical-based synthetic drugs. In this review, we have discussed the possible role of AGL as a promising herbal-chemo remedy during human diseases, viral infections and as an immunity booster.

## 1. Introduction

*Andrographis paniculata* (AP) a therapeutic herb, has been discovered and practiced as an effective herbal immuno-drug in traditional medicine systems to cure several health disorders worldwide [1]. Various scientific research has been conducted on the molecular and biochemical aspects of AP to improve the biosynthesis of its active ingredients i.e., andrographolide (AGL) [2]. The strong effects of AGL alike toxins, insecticides interfering with molting hormone pools-controlled insect pests thereby saving food crops with global economic benefit [3,4,5]. A decade of proliferating scientific research with AGL based on the active components established its multifarious pharmacological effect as an anti-bacterial, anti-viral, and anti-inflammation [6,7]. Till today the role of AP and AGL in human health has been explored and a long list of acute and chronic illnesses like infertility, diarrhea, ischemia, pyrogenesis rheumatoid arthritis, obesity, upper respiratory tract infection, fever, hepatic and neural toxicity, cancer, etc., [8,9] signifies its role with relieving treatment history. Apart from many beneficial effects, AGL (in excess) may have harmful effects, but several scientific studies already focusing on such issues provided adequate data that it is still relatively safe. One such study with mice showed that a single oral AGL administration at a very high dose (2000 mg/kg) was not found to induce mortality despite altering the total body and organ weight, while no inflammatory responses and changes in hematological parameters were recorded [10]. It is also possible to administer AGL inside the cell using a carrier to further test its efficacy in cell culture and possibly to detect direct effects as mentioned in Sinha et al. [11] where the TaRKD-TALE protein was delivered in wheat microspores using a cell-penetrating peptide and expression regulation of microspore embryogenesis associated genes were monitored. This provides a reason for AGL to become a new scientific focus for researchers who intend to investigate its beneficial role in multiple diseases (mild, acute, and chronic) prevention, suppression, and therapy.

The therapeutic effect of AGL was tested by experimental and clinical research, which proved effective in overcoming microbial infections. Hua et al. [12]. showed that AGL inhibits the growth of *C. trachomatis* bacteria which cause sexually transmitted disease and protects tissue from inflammation and damage. It significantly reduced the secretion of IL-6, IL-8/CXCL8 and interferon-γ-induced protein 10 being produced by the *C. trachomatis* infected host cells. Likewise, other researchers have also shown the inhibitory and suppressive effects of AGL on viruses such as human immunodeficiency virus (HIV) [13], herpes simplex virus type 1(HSV-1) [13,14], hepatitis B (HBV) [15], hepatitis C (HCV) [16], and influenza virus [17]. Similarly, AGL was shown to be effective as an anti-viral agent against the Zika virus [18] and Chikungunya [19]. AGL has been used in large amounts to boost up immunity during viral outbreaks worldwide including the Indian dengue outbreak in 2006, where it was proved effective with a decrease in (+ve) cases and infection. The hypothesis was supported by the sequential in vitro studies through quantification of Dengue (DENV) inhibition being induced by AGL application [20]. Similarly, Ramalingam et al. [21] applied maximum nontoxic AGL dose and showed most of the antiviral inhibitory effects with DENV 1-4-infected Vero cells. Recently, Li et al. [18] evaluated the anti-viral activity of AGL against the Zika (ZIKV) and Dengue (DENV) viruses and confirmed their potential to be developed as an anti-ZIKV, anti-DENV herbal agents. Moreover, Kaushik et al. [22] used in vitro and in silico studies to characterize the isolated compounds from AGL with its active anti-dengue property against DENV-2.

Focusing on COVID-19, in silico study of Enmozhi et al. [23], Sharma et al. [24], proved that AGL functioned as a potential inhibitor of SARS-CoV-2 main protease. It also showed to have better inhibitory properties of proteases than other proposed inhibitors [25,26]. In various other examples, AGL and its derivatives have been used to successfully treat pediatric pneumonia and upper respiratory infections. While its combined application with other anti-inflammatory agents decreased the production of pro-inflammatory factors and cytokine secretion resulting in curative effects on upper respiratory tract infections [7]. AGL promoting the body’s immunity to achieve its curative effect against respiratory inflammation and *Shigella dysenteriae* is also well documented [27]. With such background in the present review, we aim to focus on the recent updates and the broad-spectrum effectiveness of AGL concerning human health specifically communicable diseases, microbial infections, immunity enhancement to fight against the viruses like COVID-19 and SARS-CoV-2. While the future prospective of AGL and its components as an effective chemo-herbal drug will also be discussed.

## 2. Functional Prospective of Andrographolide (AGL) on Human Health

**Figure 1:**
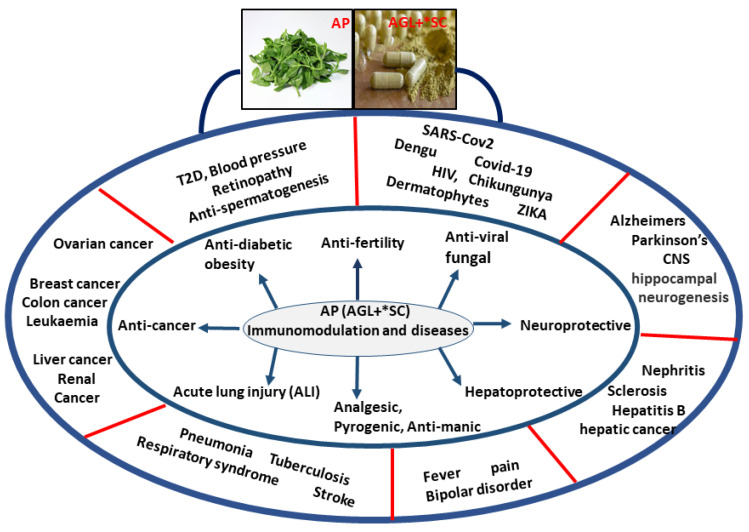
Possible role of *Andrographis paniculate* (AP) in medicine and human diseases. The disease prevention using andrographolide (AGL) is the combined effects of its anti-inflammatory, anti-oxidative, anti-pyrogenic, and immunomodulatory properties. *SC = synthetic compound.

In an immunocompromised patient, AGL intake plays a very important role by primarily initiating the immune response via modulating their complimentary system, granulocytes, and macrophages, which is useful in overcoming various diseases and infections (Figure 1).

It enhances the production of naturally occurring killer cells and stimulates the secretion of several cytokines and chemokines, which are released during disease progression [28]. Dynamin-related protein 1 (DRP1) identified as a target protein of AGL when bound together inhibited its GTPase activity, preventing neuronal damage and excessive mitochondrial fission during Parkinson’s disease [29]. Today studies targeting disease- specific molecular markers regulation using AGL need attention; e.g., Cordon-bleu protein like-1 (COBLL1) serves as a molecular marker for the patient’s overall survival in chronic lymphocytic leukemia (CLL) and its AGL-induced possible modulation might benefit CLL therapy [30]. The cytotoxic property of AGL against many cancerous cell lines was linked to its structural identity containing α-alkylidene γ-butyrolactone moiety and three hydroxyls at C-3, C-19, and C-14. It was found to upregulate HLJ1 proteins, which are known to suppress tumor cells, induce hepatocellular cancer cell cycle arrest at the G2/M phase and ROS-mediated cell death in HepG2 cell lines (hepatocellular carcinoma) [31]. Whereas inhibition of HepG2 cell proliferation and induction of caspase-independent cell death was also reported.

**Figure 2:**
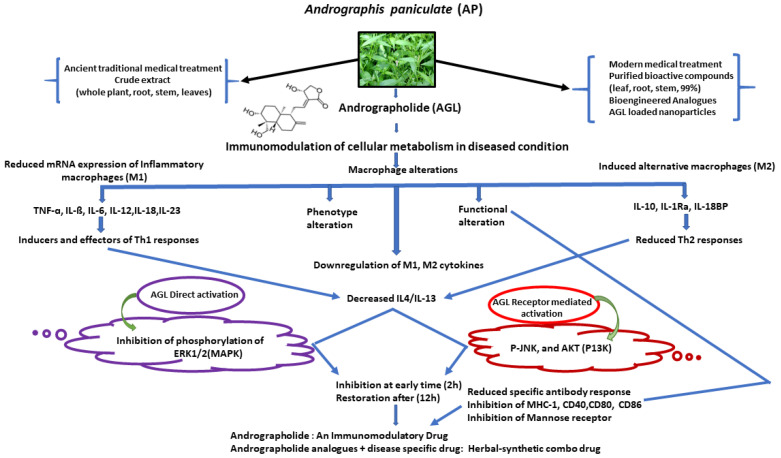
Schematic representation of the effects of andrographolide (AGL) and possible mechanism of its immunomodulatory regulation. Abbreviations: IL, interleukine; Th,T-helper cell type; MHS-1, major histocompatibility complex class II; CD, cluster of differentiation 80; CD86 cluster of differentiation; CD40, cluster of differentiation.

In carcinogenic cells, AGL regulates the epidermal growth factors, transferrin, and interferes with the nuclear factor-κB binding to neutrophilic DNA [32], thereby suppressing cancer cell proliferation, angiogenesis, and metastasis [8]. Among several properties of AGL, anti-inflammation targeting the expression inhibition of intercellular adhesion molecule-1 gene in monocyte cells activated by α-tumor necrosis factor is of great importance. Chen et al. [33] showed that the inflammatory response was associated with the decreased tumor necrosis factor-α (TNF-α) which induced intercellular adhesion molecule-1 (ICAM-1) expression and adhesion of HL-60 cells into human umbilical vein endothelial cells (HUVEC). AGL can modulate the innate and adaptive immune responses by regulating macrophage phenotypic polarization and Ag-specific antibody production where MAPK and P13K play an important role in macrophage activation and polarization. Such alteration through AGL can be achieved in different ways including the modulation of mRNA of inflammatory M1 macrophages, phenotypic alteration, functional alteration, and inducing the expression of alternative macrophages. This can cause a change in the expression of genes related to interleukins (IL4) and (IL13) which are involved in the macrophages metabolic pathways (Figure 2). The modulation of IL4 and IL13 not only affects macrophage but also ultimately affects the surrounding cells and tissues and thereby influencing the host defense and immunity. In the pathways, AGL can increase phosphorylation of p38 MAPK and inhibit the RIP2/Caspase-1/NF-jB [34]. Andrographolide application significantly reduced experimental autoimmune encephalomyelitis (EAE) symptoms in mice by impeding T-cell and antibody responses directed to myelin antigens (Figure 2). Results suggested that AGL can block T-cell activation in vitro and might be useful in the modulation of detrimental T-cell responses [35].

Exploring the beneficial effect of AGL on diabetes considering its worldwide propagation has always been of key priority. Diabetes-oriented research study showed that AGL scavenges the reactive oxygen species (ROS) and reduces the phenotypes of diabetic nephropathy (DN) in high glucose cultured MES-13 cells by intracellularly regulating the signal transduction pathway. Zhang et al. [36] showed that the application of AGL prevented the development of diabetes in autoimmune diabetic mice via strengthening its immune tolerance. Recently, a very interesting study by Su et al. [37] stated that AGL lowered the glucose effect via strengthening the function of the intestinal barrier, increased the A. *muciniphila* microbial species and prevented diabetes-type 2 (T2D). Studies with mice model helped explore AGL regulated glucose mechanism enormously [38].

With the advancement of therapeutic technologies, AGL nanoparticles (ANPs) are being in limelight today with their extraordinary adaptability, compatibility, bioavailability, and surprising loading capacity of the drugs. Undergoing constant modifications in their generation they were found to possess hepato-protective, anti-bacterial anti-malarial (anti-plasmodial), and anti-viral properties [39,40]. The increased anticancer efficiency of ANPs was found by Roy et al. [41] in mice model MCF-7 cells bearing experimentally induced *Ehrlich* ascites carcinoma. Sanati et al. [42] reported successful inhibition of neuroblastoma and cervical cancer cells by nano-encapsulation of the rich extracts of AGL. Further, AGL-loaded solid lipid nanoparticles inhibited head and neck cancer [43]. Andrographolide-loaded nanocochleates were assessed for their physiochemical properties to be used as an oral delivery alternative for clinical trials in cancer therapy [44]. In addition, AGL nanoparticles were also tested and established to protect against cigarette smoking-induced chronic obstructive pulmonary disease [45]. It controlled aggressive asthma, where the pulmonary administration was reported to be more effective than oral administration [46]. Therefore, abovementioned functional prospects of AGL and its nanoparticles could be a boon to modern therapeutic practice with the possibility to participate in being a future herbal-chemo drug.

Andrographolide sulfonate (Andro-S) one of the derivatives of AGL is used in the treatments of inflammation-related diseases, however, Andro-S is effective in reducing acute lung injury (ALI). It was analyzed using an iTRAQ-based quantitative proteomics approach and by immunohistochemistry analysis [26]. The bioinformatic analysis of Gao et al. [26] revealed the inhibitory effects of Andro-S on lipopolysaccharide (LPS) induced ALI using potential targets as neutrophil elastase (ELANE), cathepsin G (CTSG), myeloperoxidase (MPO), and other three neutrophil-derived proteases. In this study, they assumed that the possible mechanism was by attenuating the expression of neutrophil-derived proteases, which may play a crucial role in the recovery of ALI. Such studies support a similar protective effect of Andro-S in reducing the ALI effect of Coronavirus (COVID-19) known to primarily target the respiratory tract.

### Effectiveness of Andrographolide (AGL) with New Emerging Viruses and Their Frequent Mutant Counterparts (SARS-CoV-2 and COVID-19)

Recent pandemics with SARS-CoV-2 and COVID-19 accelerated studies targeting human immunity and defense against constantly emerging novel viruses. The severity of such pandemic accelerated grant funds from the World Health Organization (WHO), the European Union (EU), various governmental and research organizations supporting scientific research to develop effective drugs and vaccines capable of reducing worldwide infections and fatalities (October 2021, 4.96 M).

Development of new experimental methodologies is also in progress, where Chen et al. [47] constructed and validated SARS-CoV-2 drug target protein microarray and hypothesized it as a useful tool for pharmacological study. Khanit et al. [48] demonstrated AGL-induced anti- SARS-CoV-2 activity using Calu-3-based anti- SARS-CoV-2 plaque assay, where the effectiveness of AGL in SARS-CoV-2-infected Calu-3 cells of lung epithelial tissues was determined and AGL application significantly inhibited the production of infectious virions (0.034 µM; IC50—0.036 µg/mL). Researchers also advanced using molecular modeling and docking tool focusing on modulation of the immune system by inhibiting SARS-CoV-2 virus interaction with cellular receptors and thereover blocking NFkB1 and TNF pathway and restricting the COVID-19-induced cytokine storm responsible for the organ damage and mortality [49]. One such study showed that AGL exhibits binding affinity toward spike glycoprotein of SARS-CoV-2 and ACE2 receptor and therefore should be functionally explored as a therapeutic, prophylactic agent for restricting viral and host cell interactions [50]. Interestingly, Shi et al. [51] reported inhibition of main 2019-nCoV and SARS-CoV-2 proteases by covalent linkage with the application of AGL and its fluorescent derivatives.

With the background of 3D complex structure, SARS-CoV-2 S protein ectodomain binding to human ACE2 peptidase domain focusing on ACE2 inhibitors showed that AGL induced ACE2 receptor inhibition and its binding with the S protein of SARS-CoV-2 [52]. While Alazmi and Motwalli [53] proposed two natural origin S protein inhibitors (AGL and Pterostilbene) displaying better ACE-2 receptor and SARS-CoV-2 S protein binding potential thereon proving its beneficial property in attenuating the viral outbreaks. Further, Rajagopal et al. [54] through in silico approach explored the binding modes of AGL with the active site of SARS-CoV-2 main protease. Li et al. [55] using network bioinformatics analysis based on clinical knowledge prioritized AGL (among 30 candidates) as a potentially effective COVID-19 repurposable drug. Interestingly, Zhang et al. [56] in his clinical trial showed that injection with Xiyanping (XYP), a Chinese herbal compound (composed of 9-dehydro-17-hydro-andrographolide and sodium 9-dehydro-17-hydro-andrographolide-19-yl sulfate) improves and recovers COVID-19 patients having mild and moderate symptoms. Today, advancement with the laboratory and pharmacological studies focusing on AGL as a herbal bio-active compound has accelerated huge verified data with scientific outputs establishing its potential immune protective role in combating such viral outbreaks.

## 3. Modification of Andrographolide (AGL) and Its Constituents: Semi-Synthetic Antiviral Compounds

Bioengineering of active phyto-molecules and their components for human health benefits are the most emerging concept in the development of a herbal-chemo drug. Purposefully, the core structure of AGL was targeted and modified at various parts with emerging trends i.e., new structural features fitted to the receptors, the substitution of metabolically active compounds i.e., lipoic acid increasing the ability of the designed analog to reach the targets and induce selective biological activities thus, acting as pro-drugs. These modified analogs and derivatives were further in vitro tested for the treatment and prevention of a wide range of viral infections having mentioned below.

AGL and its constituents has been intensively investigated and established for antiviral activities against multiple viruses causing diseases including COVID viruses MERS-CoV, SARS-CoV, and flaviviruses [57,58,59]. 14-DDA, the most prominent analog of AGL was found to be active against several viral infections [60,61]. With modifications at C-3 by substituting OH to NOH and amides, a higher therapeutic index (TI) with moderate activity was recorded in comparison to the parent compound. 13,14-dihydroandrographolide alteration with g-lactone to N-methyl-g-lactam was highly effective against HIV, giving almost half activity of AGL with twice increase in the TI. Interestingly, the potency was found to be lost with the removal of N-methyl on the g-lactam. Whereas the best tested analog for anti-HIV activity was 3,19-di (acetoxy-benzyl)-isoandrographolide having TI more than 51 [62].

Many other constituents of AGL were found to be effective as an anti-viral and in prevention of the pre-infection like 12 and 14-acetylandrographolide, 14-deoxy-11, 14-acetyl-3,19-isopropylidenyl andrographolide, and 3,14,19-triacetyl andrographolide etc., [63,64]. Some of the AGL analogs and its derivatives with their activity level (CC_50_, EC_50_, SI/TI) on selective cell lines against the target viruses are shown in Table 1.

The corresponding oxime of 3-keto derivative, andrographolic acid amide derivatives, and 12-ester of 12-hydroxy-14-deoxy-13,14-dehydroandrographolide were tested against HIV with positive results [69]. Some modified analogs like 14-a-Lipoylandrographolide were effective against H9N2, H5N1, and HINI influenza as it inhibited viral adsorption into red blood cells by blocking cellular receptor bindings and interfering with viral haemagglutinin [17]. Hepatitis B virus (HBV) was successfully treated with the esterified derivative of 14-DDA at C-19 with pyridinecarboxylic acid or 2-thiophenic and furoic acid by inhibiting DNA replication, HBsAg and HBeAg antigens [15]. During the anti-influenza virus study against H3N2, the benzyl amino derivative, 19-dihydroxyl-17-(*N*-benzylamino)-7, 13-ent-labdadien-15, 16-olide showed the greatest potency being 1.5-fold more potent than the parent AGL [69]. When tested for anti-HBV activity with the structure–activity relationship the conjugated double bonds between C-11 and C-14 or C-12 and C-15, heterocyclic aromatic moieties and the free -OH was highly effective [15]. Genes and proteins responsible for viral replications were found to be influenced by 3,19-isopropylidenyl andrographolide, which is probably due to the structural similarity with an anti-DNA replication compound 1,3-dioxolane [70]. Esterification of 11,12-didehydroandrographolide with succinic acid improved the activity against hepatitis C and H5N1 infections [71]. Today, AGL and its constituents are tremendously examined and getting established as semi-synthetic antiviral compounds with potential therapeutic effects.

## 4. Prospective of Herbal-Chemo Drug Development: A Potential Approach for Human Immunity Enhancement

The future of advanced pharmacological research should be confined to designing herbal supplemented synthetic chemo-therapeutic agents, which may prove not only immunosuppressive but also be combo-safe and can enhance molecular bioavailability and efficacy for better therapeutic outputs. Therefore, the identification of drug targets for AGL and its bioengineered components are of prime interest. Moreover, drug determination in biological samples is crucial to determine safer dose limits during effective drug development and discovery. Today the methods of separation, quantitative estimation of andrographolide (AGL) from a various herbal, complex mixture, and biological sources are established using spectrophotometry, chemiluminescence, electroanalytical and chromatography techniques [72] and being helpful in accurate detection and determination of its functional impact (Table 2).

Several pharma companies used herbal substances (e.g., curcuma, asafoetida, AGL) for drug product development, manufacturing, and executing the clinical trial development for various formulations. In this regard many Chinese formulas containing AP, *AGL* have been developed and patented as medicines with various formulations and drug dosages against the active viruses (www.cnki.net, accessed on 20 November 2021). Moreover, modulation of the immune response of these combo’s (herbal-synthetic agents) and their interaction with specific receptors and cellular components are being actively studied, monitored, and the data reveal the addition of their beneficial application [94]. Drugs like Remdesivir, Hydroxychloroquine, Captopril, Nafamostat in combination with AGL constituents are established to have a good antiviral activity targeting ACE-2 receptor and spike protein complex, RdRp, PL^pro^, 3CL^pro^, N-protein RNA-binding domain of SARS-CoV-2 [95,96,97,98].

AGL with its therapeutic potential proves to be the most fitted herbal-chemo drug candidate having most of the therapeutic indices in various disease treatments. Previously, Basak et al. [99], showed the role of a new combination of drugs using the AGL- derived natural product “Restomune” in HIV management. Restomune, a natural product was approved by Health Protection Branch (HPB), Govt. of Canada (DIN 00774448) for the relief of colic/gastrointestinal gas disorder. The case report says that in some HIV cases Restomune as an alternative medicine boosted up the immune system by restricting the HIV reproduction and in combination with multiple RT-inhibitor such as AZT (Zidovudine or Retrovir from Glaxo Wellcome) or DDI (Didanosine, Videx from Bristol-Meyers Squibb), antioxidants, vitamins, which are much affective in the modulation of CD4 (+) T lymphocytes by viruses and finally preventing oxidative stress organ damage. It was also reported to promote CD4 (+) T-cell growth, which may compensate for the loss of CD4 (+) T cells at the time of fatal viral infection. In addition, the drug Restomune was also tested with a combination of other HIV- specific drugs and resulted in the rapid recovery of CD4 (+) T-cell counts. This combo-drug Restomune acts as an immune drug and significantly reduces and enhances the immune system and SARS-CoV-2 [94,96]. Additionally, the examples of AP-based drugs and their active principles being successfully marketed are listed in Table 3. These combo multitargeted drugs benefit human health by relieving seasonal cold, fever, strengthening immunity, liver ailment, respiratory support, cardiovascular support, etc.

## 5. Conclusions

In general, this review summarizes the therapeutic role of andrographolide (AGL) in boosting human immunity and treating diseases. The protective role of this multitarget bioactive herbal compound has long been established for treating microbial infections, fatal diseases like cancer, diabetes, acute respiratory tract infections, etc. AGL’s bioengineered analogs and derivatives with improved solubility, bioavailability, and therapeutic index (TI) is constantly synthesized and scrutinized providing directions for future therapeutic benefits. During the current global COVID-19 pandemic a lot of AGL-based productive research is on the go, yet a lot awaits to be explored at its target molecules having immune-modulatory and anti-viral properties which is crucial for drug designing. Sensitive technological advancements in AGL quantification methods from various commercial and biological sources may serve as the key factor in the effective dose assessment during drug development and disease therapy. This review article proposes that AGL and its constituents have a future for combo herbal-synthetic drug and may facilitate the pharmaceutical industry to design, validate, and produce AGL-based synthetic drugs targeting biological pathways for multiple therapeutic choices thereby increasing vitality and immunity.

## Figures and Tables

**Table 1:** AGL analogs and derivatives with their activity level (CC_50,_ EC_50,_ SI/TI) on selective cell lines, cytotoxicity assay against target viruses.

AGL Analogues ^$^/ Derivatives ^#^	Virus	Targeted Cells	CC_50_ (µM)	EC_50_ (µM)	Cytotoxicity Assay	Selective Index (SI)/Therapeutic Index (TI)	Reference
14-aryloxy analogues ZAD-1 ^$^	ZIKV DENV		136.3 ± 6				[18]
			510.3 ± 53	27.9 ± 1.7		9.8 (SI)	
			272.9 ± 22	22.6 ± 1.8		6.6 (SI)	
			148.8 ± 40				
		BHK-21 Vero					
14-aryloxy analogues ZAD-2 ^$^			217.7 ± 16	-	MTT Assay	-	
			179.2 ± 13				
			194.2 ± 17				
			196.8 ± 7				
		A549 HEK293T/17					
14-aryloxy analogues ZAD-3 ^$^			175.0 ± 7	-		-	
			186.1 ± 25				
			190.1 ± 22				
			201.4 ± 25				
	Human immunodeficiency virus HIV-1					23.74 (TI)	[65]
3-*O*-Nicotinoyl-19-*O*-(*n*-decanoyl)-dehydroandrographolide ^#^			103.5 ± 128.73	82.89 ± 7.33			
						34.07 (TI)	
3-*O*-Nicotinoyl-19-*O*-(1-naphthalene acetyl)-dehydroandrographolide ^#^			>200	5.87 ± 1.96		18.02 (TI)	
3-*O*-Nicotinoyl-19-*O*-phenylacetyldehydroandrographolide		C8166	>200	11.34 ± 2.56	MTT Assay	16.8 (TI)	
3-*O*-Nicotinoyl-19-*O*-(3,4-dimethoxyphenylacetyl) dehydroandrographolide			>200				
3-*O*-Nicotinoyl-19-*O*-(3,4-dimethoxyphenylacetyl) dehydroandrographolide			>200	11.76 ± 3.66			
14-deoxyandrographolide (DAD) ^$^	Herpes simplex virus type 1 (HSV 1)		80				[14]
3,19-isopropylideneandrographolide (IPAD) ^$^			40				
3,19-dipalmitoylandrographolide ^$^			4.2	-		-	
14-acetyl-3,19-isopropylideneandrographolide ^$^		Vero	5.9		Anti-HSV-1 assay		[64]
3,14,19-triacetylamdrographolide ^$^			6.4				
14-dehydroxyandrographolide- 12-sulfonic acid sodium salt (DASS) ^#^	H9N2 H5N1 H1N1	MDCK	3720 ± 725	142.2 ± 10.3	MTT Assay	26 (SI)	[17]
				222.9 ± 14.9		17 (SI)	
				171.1 ± 13.5		22 (SI)	
14-a-lipoyl andrographolide (AL-1) ^#^			785 ± 330	8.4 ± 2.4		93(SI)	
				15.2 ± 4.09		51(SI)	
				7.2 ± 1.5		109(SI)	
19-*O*-(3′, 4′, 5′-Trimethoxy) cinnamoyl dehydroandrographolide ^#^	Hepatitis B (HBV)	HepG2 C8166	>1706		MTT Assay	165.1 (SI)	[15]
19-*O*-(2′-Thenoyl)-14-deoxy-14,15-didehydroandrographolide ^#^			2466			104.9 (SI)	
19-*O*-Nicotinoyl-14-deoxy-14,15-didehydroandrographolide ^#^			2054	-		126.0 (SI)	
19-*O*-Cinnamoyl dehydroandrographolide ^#^			183			15.5 (SI)	
3,19-(30-Nitrobenzylidene)-andrographolide ^#^	Human immunodeficiency virus (HIV)		745	-	MTT Assay	1460 (TI)	[66]
14-(20,60-Dichloronicotinoyl) ester of andrographolide ^#^		TZM-bl cells	10354			12,474 (TI)	
(14α)-(Quinolyl-5′,7′-dichloro-8′-oxy)-19-acetoxyandrographolide ^#^	ZIKV	SNB-19 Vero	88.7 ± 1.1	4.5 ± 0.2	ZIKV titer assay	19.7 (SI)	[67]
			85.0 ± 1.6			18.9 (SI)	
14β-(8′-quinolyloxy)-3,19-diol ^#^			22.7 ± 1.1	1.3 ± 0.1		>16	
			20.8 ± 0.5				
(14β)-(Quinolyl-5′,7′-dichloro-8′-oxy)andrographolide ^#^			>100	13.3 ± 0.5		>7.5	
(14α)-(Quinolyl-5′,7′-dichloro-8′-oxy)andrographolide ^#^			85.2 ± 1	7.8 ± 0.4		10.9	
			82.5 ± 2.2			7.5	
3,19-isopropylideneandrographolide (IPAD) ^$^	HSV 2	Vero	39.71	-	Anti-HSV-1 assay	2.20 (SI)	[18,68]
	HSV 1-KOS					2.34	
	HSV 1-dxpIII					2.32	

Values with (±) represents the mean ± SD of three determinations. CC_50_ represents the minimum cytotoxic concentration that caused the reduction of viable cells by 50–80%. EC_50_ represents the minimum inhibitory concentration that reduced the cytopathic effect by 50%.

**Table 2:** Advancement in methods for quantitative estimation of andrographolide (AGL) from various herbal, multi-complex mixtures, and biological sources.

Source	Method/Instrument	Solvent/Instrument Detection	Limit of Detection (LOD)/Limit of Quantification (LOQ)	Reference
*A. paniculate* bulk powder	UV/VIS Spectroscopy	Methanol: water (50:50 *v*/*v*)		[73]
Herbs and herbal formulations		95% ethanol, methanol, aqueous sodium hydroxide, picric acid, Baljet reagent dinitrobenzoic acid and KOH solution	1.2/4.23 μg	[74]
Andrographolide in plant material	FT/IR spectroscopy	FTIR single reflectance horizontal ATR cell spectrometer (Perkin Elmer)	1.0/3.34 μg/mL	[75]
Andrographolide Powder		Perkin Elmer spectrometer with KBr Optics and mercury cadmium telluride A detector	1.5/15 μg	[76]
Andrographis tablets (Commercial)	Flow-injection chemiluminescence	Chemiluminescence analysis system (IFFM-E mode flow injection) LC-20 A HPLC, fluorescence spectrophotometer, UV/VIS spectrophotometer	0.0742 μg/mL	[77]
Human plasma (Andrographolide treated)	Cloud Point Extraction (CPE)	Triton X-114 (5%, *v*/*v*), 0.45 g NaCl, Agilent 1100 liquid chromatograph M.P.-methanol-acetonitrile-0.5% formic acid aqueous solution (40:17:43, *v*/*v*/*v*), SB C18 column (5 μM)	0.032 μg/mL	[78]
Human urine (Andrographolide treated), *A. paniculata* oil extract	Pulse Voltammetry Measurements	Autolab Pgstat 302 N Electrochemical system, Methrom Autolab B.V. (The Netherlands), pH meter, Boron-doped diamond electrode	0.75 μM	[79]
		Heptane (0.81%, *w*/*w*), SDS (3.31%, *w*/*w*), butan-1-ol (6.61%, *w*/*w*) Sodium tetraborate buffer (10 mM), pH 9.2. MDQ CE instrument (PDA detector)		
Andrographolide (standards), dehydro-andrographolide commercial tablets.	Microemulsion electrokinetic chromatography (MEEKC)		0.30 and 1.0 μg/mL	[80]
Andrographolide (standards), dehydro-andrographolide commercial tablets.	*Micellar Electrokinetic Chromatography* (MEKC)	SDS (15 mM) in 30 mM borate buffer pH 9.5. Waters (Milford, MA, USA) fixed wavelength with UV detector Quanta 4000E CE system	8.64–60.1 mg/L	[81]
*A. paniculate (plant material)*	High-speed Counter-Current Chromatography (HSCCC)	Multilayer coil counter-current chromatograph (Potomac, MD, USA), Water/methanol/ethyl acetate/n-hexane (2.5:2.5:4:1) 1.5 mL/min	-	[82]
Herbal extract and multi-herbal formulations,	High-performance Thin-layer Chromatography (HPTLC)	Silica gel 60 F254 Coated TLC Aluminium plates. Chloroform:Toluene:Methanol (66:26:8, *v*/*v*/*v*).	3.5 and 11.7 ng	[83]
Polyherbal Livogat capsule,		Toluene:Acetone:Formic acid (9:7:1, *v*/*v*/*v*)	62.91 and 209.7 ng per spot	[84]
Bulk Drug and *A. paniculata* formulations determination.	High-performance liquid chromatography (HPLC)	Dichloromethane-Toluene-Ethanol (6:3:1, *v*/*v*/*v*),	34–109 and 112–363 ng per spot	[85]
Andrographolide in Self-Nano Emulsifying Drug Delivery System (Snedds)		Isocratic methanol:Water (70:30) 0.8 mL/min, Xterra MS C 18 column (150 mm × 4.6 mm, 5 μm)	1.95 and 3.13 μg/mL	[86]
*Andrographolide hypophyllanthin* and *phyllanthin* (herbal liver protective formulations)		Gradient-0.1% orthophosphoric acid (sol. A) and (1:1) acetonitrile: methanol (sol. B), Symmetry C8 column (250 mm × 4.6 mm, 5 μm)	20 and 60 ng	[87]
Toxiroak Premix (Polyherbal mycotoxin inhibitor)		Isocratic acetonitrile:ortho-phosphoric acid (0.1%), 40:60 *v*/*v* 1.0 mL/min, C18 column Phenomenex luna (250 mm × 4.6 mm, 5 μm)	0.06 and 0.2 μg/mL	[88]
Rat whole blood administered with Andrographolide containing liposomes and commercial tablets		Isocratic methanol:Water (52:48 *v*/*v*) 0.8 mL/min, Chromasil ODS Column (25 mm × 4.6 mm, 5 μm)	0.015 and 0.053 μg/mL	[89]
Urine and faeces of New Zealand rabbit’s (23187-INDUCED and treated with andrographolide)		Isocratic methanol:water (55:45) 1 mL/min 0.5 mL/min, C 18 column (250 mm in, 5-μm and 120 Å pore size)	1.87 and 5.45 μg/mL	[90]
*A. paniculata* fresh leaves and stem	Hyphenated technique	Gradient A:0.1% formic acid in water (B) 0.1% formic acid in acetonitrile 0.3 mL/min, Acquity BEH C18 (2.1 mm × 50 mm, 1.7 μm)	0.18 and 0.75 ng/mL	[91]
Pharmaco-kinetic analysis and distribution of andrographolide in rat tissues		Gradient-2 mM ammonium acetate buffer (A), mixture of acetonitrile and solvent A (80:20, *v*/*v*) (B) 0.8 mL/min, C18 column (2 mm × 30 mm, 5 μm)	3.91 ng/mL (LOQ)	[92]
Human plasma determination of four major active diterpenoids from *A. paniculata*		Gradient: water (A) acetonitrile(B) 0.5 mL/min, Kinetex column (4.6 mm × 150 mm, 2.6 μm)	2.50 ng/mL (LOQ)	[93]

**Table 3:** Bioactive extract of *Andrographis paniculate* (AP), its active principles from different commercial sources and their proposed functions on human health.

Company/Manufacturer	Drug/Supplements	Composition of the AGL Based Combo-Drug’s Active Principles	Proposed Functional Role
EU Nature	Armor2 Andrographis Pure 800 MG	AP (Plant extract) 600 mg, 10% Andrographolides (200 mg), Gelatine	Strengthens immunity, seasonal protection, cold and flu
https://ecosupplements.eu/product/eu-natural-amor-2-andrographis-800mg-60-vcaps/ (accessed on 20 November 2021)			
Bixa Botanicals	Andrographis	AP (Plant extract) 450 mg, 20% AGL, Gelatin	Fever, liver ailments, blood sugar control, headache, anti-inflammation
https://bixabotanical.com/search?collection=all&type=product&x=0&y=0&q=Andrographis (accessed on 20 November 2021)			
Nine Life	Andrographis	Andrographis 900 mg, Gelatin, Rice Powder	Supports healthy immune and liver function
https://www.ninelife.eu/search?type=product&q=andrographis (accessed on 20 November 2021)			
Terry Naturally Vitamins	Andrographis and Ashwagandha	AP (leaf extract) 200 mg, Ashwagandha (leaf and root extract) 150 mg, Hydroxypropyl methylcellulose, microcrystalline cellulose, magnesium stearate, silica, maltodextrin	Immune defence, antistress, energy and endurance, focus and clarity
https://www.terrynaturallyvitamins.com/andrographis-and-ashwagandha (accessed on 20 November 2021)			
Terry Naturally Vitamins	Andrographis EP80^TM^ immune	AP (Leaf extract) 60 mg, Melatonin 5 mg, Selenium 65 mcg and Zn 15 mg	Upper respiratory function support, cellular level support, restorative sleep
https://www.terrynaturallyvitamins.com/andrographis-immune (accessed on 20 November 2021)		Hydroxypropyl methylcellulose, microcrystalline cellulose, silica, cellulose powder, citric acid	
Terry Naturally Vitamins	Andrographis EP80^™^ Extra Strength	AP (Leaf extract) 40 mg, Hydroxypropyl methylcellulose, microcrystalline cellulose, magnesium stearate, silica	Immune function and upper respiratory tract health, joint health, daily energy and adaptability, intensive cellular health and DNA protection from oxidative stress, mental clarity and brain function
https://www.terrynaturallyvitamins.com/andrographis-ep80-extra-strength (accessed on 20 November 2021)			
Peak performance	Andrographis +Echinacea Vegan Capsules	AP (aerial extract) 200 mg, *Echinacea purpurea* root 200 mg	Immune support, stress relief respiratory support
https://www.ninelife.eu/products/andrographis-echinacea-vegan-capsules-made-with-organic-echinacea-pure-andrographis-paniculata-nilavembu-green-chiretta-complex-from-natural-plant-extracts-immune-respiratory-support-pills?_pos=34&_sid=b865e717f&_ss=r (accessed on 20 November 2021)			
Swanson	Full spectrum *Andrographis paniculata*	AP (aerial parts) 400 mg, Gelatin, microcrystalline cellulose, magnesium stearate, silica	Immune system support
https://ecosupplements.eu/?s=Full+spectrum+Andrographis+paniculata&post_type=product (accessed on 20 November 2021)			
One planet nutrition	Nano Andrographis	Aqueous nano Andrographis 250 mg, rice flour	Immune support, superior absorption, and bioavailability
https://www.oneplanetnutrition.com/shop#!/Nano-Andrographis-120-Caps-250-mg/p/192856679/category=27372415 (accessed on 20 November 2021)			
Solaray			
https://solaray.com/products/andrographis-aerialextract?_pos=2&_sid=aeaccba57&_ss=r (accessed on 20 November 2021)	Andrographis aerial extract	AP (aerial extract) 300 mg, AGL 4%	Immunity booster, respiratory tract benefit
Natur’s Way	Andrographis	AP (stem, leaf, flower) 100 mg, 10% AGL	Immune support, seasonal protection against cold and flu
https://www.iherb.com/search?kw=Andrographis (accessed on 20 November 2021)			
Cardiovascular Research	Restenoril Andrographolide	AP (stem) 500 mg, Gelatin, Methocel, Potassium sorbate	Supports a healthy immune response, promotes healthy cardiovascular function, provides antioxidant support
https://www.nhc.com/restenoril-andrographolide-by-cardiovascular-research (accessed on 20 November 2021)			
Herbadiet	Andrographis Extract	AP (leaf extract) 500 mg	Liver detoxification; immunity booster; fights cold and flu
https://herbadiet.in/search?q=Andrographis+Extract (accessed on 20 November 2021)			
Piping Rock	AP Extract	AP (stem) 400 mg, Rice Powder, Gelatin Capsule, Vegetable Magnesium Stearate, Silica.	Immune support and liver function, dietary supplement
https://cz.pipingrock.com/andrographis?keywords=Andrographis%20Paniculata%20Extract&qid=1600435411 (accessed on 20 November 2021)			
Oriental Botanicals	ViraForce	AGL 62.5 mg in combination with Olive leaf 1.25 g, Honeysuckle flower bud 1 g, Echinacea root 750 mg, vitamin C (250 mg), Zinc (8 mg).	Immunity against viral and bacterial infections, common cold, influenza (flu), tonsillitis and sinusitis, fever and headache, sore throat,
https://www.orientalbotanicals.com.au/products/viraforce (accessed on 20 November 2021)			
Fusion Health	ActiViral	AP leaf 4.5 g (ai 62.5 mg) combined with olive leaf (1250 mg), oleuropein (30 mg), Honeysuckle flower bud (1000 mg), Echinacea root 750 mg, Vitamin C (250 mg), Zinc glycinate (equiv. to 8 mg)	Antiviral and immune support during infections. Multiple disease and infections.
https://www.fusionhealth.com.au/products/activiral (accessed on 20 November 2021)			
Sears	Andrographis	AP plant extract 500 mg combined with wood pulp, Colloidal silicon dioxide, Magnesium stearate, Dicalcium phosphate, Sodium benzoate	Liver support, multiple disease and infections.
https://www.sears.com/search=Andrographis (accessed on 20 November 2021)			
Panaxea International	AntiVirii	AGL (95%), Taraxasterol (20%), Chlorogenic acid (25%), Lonerica Japonica (contains: standardized Chlorogenic acid 25%)	Boosts immunity, influenza, virus, COVID-19, *SARS-CoV-1, SARS-CoV-2,* anti-inflammatory properties, pulmonary protective
https://antivirii.com/ (accessed on 20 November 2021)			
NHR Science	Andrographis	AP (leaf extract) 300 g; (Bioactive14-Neo-Andro Compound) Vegetarian capsules (Hydroxypropyl Methylcellulose), Non-GMO Rice Flour	Healthy inflammatory response, maintains bone mass and strength, immune response, supports nose, throat, and respiratory health
https://nhrscience.com/products/paractin-andrographis-paniculata-leaf-extract (accessed on 20 November 2021)			
MediHerb	Andrographis Complex. Herbal blend of chinacea root, Holy Basil leaf, and AGL	Calcium, Echinacea root 4:1 extract from Echinacea angustifolia root 500 mg; Holy Basil herb 5:1 extract; from *Ocimum tenuiflorum* herb 500 mg; AGL herb 10:1 extract from AP herb 1.0 g Containing AGL 10 mg; Holy Basil (*Ocimum tenuiflorum*) herb essential oil	Boosts immunity, supports healthy respiratory system function, helps in maintaining body temperature, encourages adaptive response to occasional everyday stress, promotes healthy liver function
https://www.natures-source.com/45126-mediherb-andrographis-complex-60tabs.html (accessed on 20 November 2021)			
Life extension^®^	Immune Protect with PARACTIN^®^	Vitamin C (camu-camu extract) 50 mg; Camu-camu extract (wildcrafted berry) 250 mg; Wellmune^®^ (highly purified Beta 1,3/1,6 Glucan from Yeast) 100 mg; PARACTIN^®^ PARACTIN^®^ mixed AGL extract from different source and analogues (25 mg)	Promotes immunity, encourages macrophage activity and natural killer cell function, provides potent antioxidants as well as vitamin C
https://www.lifeextensioneurope.com/immune-protect-with-paractin-30-vegetarian-capsules (accessed on 20 November 2021)			
